# The mast cell: A Janus in kidney transplants

**DOI:** 10.3389/fimmu.2023.1122409

**Published:** 2023-02-20

**Authors:** G. van der Elst, H. Varol, M. Hermans, C. C. Baan, J. P. Duong-van Huyen, D. A. Hesselink, R. Kramann, M. Rabant, M. E. J. Reinders, J. H. von der Thüsen, T. P. P. van den Bosch, M. C. Clahsen-van Groningen

**Affiliations:** ^1^ Department of Pathology and Clinical Bioinformatics, Erasmus University Center Rotterdam, Rotterdam, Netherlands; ^2^ Department of Internal Medicine, Division of Allergy and Clinical Immunology, Erasmus University Medical Center Rotterdam, Rotterdam, Netherlands; ^3^ Department of Internal Medicine, Division of Nephrology and Transplantation, Erasmus University Medical Center Rotterdam, Rotterdam, Netherlands; ^4^ Department of Pathology, Necker Hospital, APHP, Paris, France; ^5^ Institute of Experimental Medicine and Systems Biology, RWTH Aachen University, Aachen, Germany; ^6^ Division of Nephrology and Clinical Immunology, RWTH Aachen University Hospital, Aachen, Germany

**Keywords:** mast cell (MC), kidney transplant, rejection, fibrosis, tolerance

## Abstract

Mast cells (MCs) are innate immune cells with a versatile set of functionalities, enabling them to orchestrate immune responses in various ways. Aside from their known role in allergy, they also partake in both allograft tolerance and rejection through interaction with regulatory T cells, effector T cells, B cells and degranulation of cytokines and other mediators. MC mediators have both pro- and anti-inflammatory actions, but overall lean towards pro-fibrotic pathways. Paradoxically, they are also seen as having potential protective effects in tissue remodeling post-injury. This manuscript elaborates on current knowledge of the functional diversity of mast cells in kidney transplants, combining theory and practice into a MC model stipulating both protective and harmful capabilities in the kidney transplant setting.

## 1 Introduction

Kidney transplant (KTx) recipients often experience progressive transplant injury and loss of function. Within 10 years, approximately 50% of KTx from deceased donors and 30% of KTx from living donors suffer complete graft loss (1). Although improved donor-recipient matching and better immunosuppressive drug combination therapy has resulted in a decrease of early rejection and graft loss over the past decades, late rejection and graft loss still remain a significant problem for KTx patients (2–5). While modern immunosuppression can halt an episode of acute rejection, in approximately half of all patients their graft function will not return to baseline and they remain at high risk for subsequent graft loss (6). Using the Banff classification of Renal Allograft Pathology, renal allograft rejection can be broadly categorized into T cell-mediated rejection (TCMR) and antibody-mediated rejection (AMR) (7). Both innate and adaptive immune systems are involved in graft-injury. General tissue injury initially triggers the innate immune system, potentially leading to activation of the adaptive immune system by donor or recipient innate cell antigen presentation and mediator release through interaction with T cells (8). Interstitial fibrosis (IF) results from an abundant deposition of extracellular matrix (ECM) in the tubulointerstitial compartment, eventually leading to scar formation (9). IF is a marker for graft dysfunction (10), and fibrosis with inflammation is a strong predictor of subsequent graft dysfunction and graft loss (10–12). Inflammation within areas of IF and tubular atrophy (i-IFTA) is a transitional phase between initial inflammation and tubulitis and either resolved fibrosis or chronic i-IFTA with progressive fibrosis. i-IFTA is a strong predictor of graft failure in TMCR, but a diverse gene expression pattern is witnessed, including B cell, plasma cell and mast cell transcripts (13).

Mast cells (MCs) are a critical component of both innate and adaptive immune responses, for example in allergy and anaphylaxis (14, 15), and host defense against parasites and animal toxins (16–18). MCs are also associated with various fibrotic diseases, although their exact role in fibrosis remains controversial (19, 20). To what extent MCs contribute to the formation of graft fibrosis and its relation to transplant outcome remains unclear (2). This manuscript will first focus on the current knowledge of MCs within the KTx setting, elaborating on the functional diversity of MCs in KTx. Thereafter, an integrative model of MCs in kidney tolerance and rejection will be proposed.

## 2 Current knowledge on functionality of mast cell in kidney allografts

### 21 Mast cell development and function

The exact origin of MCs remains unclear, with both a bi-potent basophil/mast cell progenitor (21) and a unique progenitor line besides the known myeloid cell line having been proposed (22, 23). MCs have a long lifespan, sometimes outlasting an entire immune response, aiding the process of clearing pathogens, including helminths (nematodes), reptile and arthropod venoms and certain tick species (16–18). MCs also partake in and help regulate host defence against viral and bacterial pathogens (24). As first responders, MCs possess sensory and regulatory functions in inflammatory processes, such as pathogen detection, mediator release, cellular and vascular tissue activation (25), antigen presentation (26) and pathogen removal (27–31). MCs mature and reside within peripheral tissues and can be found in almost all vascularized tissue, being most abundant in and around skin and mucosal surfaces (17). Furthermore, they have a different molecular expression profile depending on the tissue they reside and mature in, but share a common transcriptional MC signature of 128 genes (32). Additionally, they contain granules filled with premade mediators, including vasoactive amines (serotonin and histamine), proteoglycans, proteases (tryptase and chymase) and cytokines (33).

As innate immune cells, MCs possess toll-like receptors (TLRs), which can be activated by pathogen- or damage-associated molecular pattern molecules (25) to effect certain MC functions like mediator release, their antigen-presenting cell (APC) capabilities and interaction with dendritic cells (DCs) (34, 35) or interaction with other immune cells (18, 19). They respond to cell injury independently of TLRs through IL-33 activation and many other mediators (36). As an ‘unprofessional’ APC they can, in conjunction with DCs, fine-tune a type 2 immune response through promoting DC migration to draining lymph nodes, thereby priming an adequate T helper 2 (Th2) cell response. MCs and DCs secrete IL-10, interferons and tissue growth factor beta (TGF-β), thereby assisting regulatory T cells (Tregs) in their immune-protective actions against alloreactive T cells (37); thus, they can both activate and inhibit T cell-mediated responses (18, 34, 38).

There are two types of mast cells described in mice based on their phenotypical characteristics and their location, namely connective tissue-type and mucosal type. The first is found more often in serosal cavities, around venules and near nerves and the latter more often in the mucosa of the gut and respiratory tract (39). In the human setting, there are two main types of MCs: those that contain tryptase granules (MC_T_), and those containing both tryptase and chymase granules (MC_TC_) (40). MC_TC_ also contain cathepsin G, a serine protease similar to chymase (41). In lung tissue, MC subtype occurrence depends on its surrounding tissue; MCs around smooth muscle tissue are mostly MC_TCs_ while MCs in alveoli are more often MC_Ts_ (42). Interestingly, in the mucosa of small intestine most mast cells are MC_Ts_ and in the submucosa the MC_Ts_ are only scarcely represented (43). Differences in the type of mast cell therefor represent their function within the different microenvironments. Distribution patterns have not been studied in kidneys, but the MC_T_ is presumed to be the most prevalent in the normal tubular interstitium (44), although an MC_TCs_ count of 54% has been observed (40).

### 2.2 Mast cells and organ transplant rejection

Chronic rejection is associated with an increase in MCs within the solid organ transplant, including kidney (40, 44–47), intestine (48), lung (49), heart (50, 51) and liver (52, 53). An increase in MCs was also observed during acute rejection (50, 53–56), although not consistently (51, 52, 57). The increase in MCs could, however, be secondary to the inflammatory response of rejection, as it is related to both IF and time post-transplantation, suggesting that MCs are a marker for cumulative burden of tissue injury (58). Due to the minimal amount of data investigating mast cells numbers in transplantation in relation to time post transplantation, it is not known whether it is time dependent.

In KTx rejection, the number of MC_TCs_ is increased in comparison to native kidneys, constituting approximately 57-60% of MCs (40, 44), although a subset of patients with rejection had a low MC_CT_ to MC_T_ ratio (40). A higher total MC count as well as a higher MC_TC_ : MC_T_ ratio is related to fibrosis and rejection (40, 44). Interestingly, both the absolute and relative amount of MC_TCs_ was drastically increased in patients with poorer transplant outcome, suggestion a more potent role of chymase in rejection and IF and a phenotype switch of MC subtype in transplant disease, a phenomenon also observed in lung Tx (59).

#### 2.2.1 Mast cell recruitment and activation

Stem cell factor (SCF) is important in MC development, maturation, activation, recruitment and chemotaxis of (im)mature MCs (60–62). SCF is secreted by endothelial cells and fibroblasts (63, 64) and binds to the c-KIT receptor. It is found in soluble form (sSCF) and membrane bound form (mSCF), the latter being cleaved into sSCF by chymase (65) and matrix metalloproteinase-9 (MMP-9) (66), both of which are released by MCs. This suggests a positive feedback loop of degranulation, with chymase release resulting in more sSCF and thus increased MC recruitment (65). SCF is linked to increased MC infiltration, fibrosis and interstitial alpha smooth muscle actin (α-SMA) (63, 67), as well as tissue remodeling (68). SCF stimulation has a protective role on (c-KIT positive) tubular epithelium and kidney function after ischemia-reperfusion injury (66, 69) and can be a predictive factor of eGFR in healthy, aging kidneys (70).

IL-9 has the ability to recruit MCs and is secreted by different cell types, including Th cells, Tregs and MCs (71). Naive Th cells express IL-9 after TGF-β and IL-4 exposure, while Th2 cells expresses IL-9 after IL-1 stimulation. IL-10 and SCF exposure enhance IL-9 synthesis by MCs, resulting in a positive feedback loop (72). Finally, IgE bound antigens can induce MC chemotaxis (61, 73). Donor specific anti-HLA I and II IgE has been found in transplant studies in both mice and humans, linking it to rejection (74). Although IgE presence in the kidney transplant is much higher in AMR, non-anti-HLA IgE antibodies are also found in areas with interstitial fibrosis and tubular atrophy (IF/TA) (47).

FcϵRI is a high affinity IgE receptor, giving MCs their infamous reputation in anaphylaxis. This receptor can be highly fine-tuned depending on the type of stimulation (75, 76); stimulation is at its strongest when bound to high concentrations of IgE with high antigen affinity and proximate IgE epitopes within the antigen (33). IgE-independent activation of MCs has also been described, for example by compound 48/80 (a synthetic ‘histamine liberator’ used to study MC degranulation (77, 78)), substance P and the Mas-related G protein-coupled receptor-X2 (MRGPRX2 or MRGX2), although MRGPRX2 is presumably absent in renal mast cells (78). Finally, MC expression of high-affinity IgG receptor FCγRI has also been reported (79). Interestingly, low dose antigen exposure of MCs can result in desensitization of the FcϵRI and MC tolerance to the antigen (75). Unique to the immune system, MCs can recover and resynthesize new granules after IgE (80, 81) or compound 48/80 mediated degranulation, after which they can be reactivated again by either mechanism (82). MCs can release granules with mixed mediator contents, or specific mediators, depending on the type of activation (33). MC stimulation and exocytosis can be highly fine-tuned, with focused or ‘piecemeal exocytosis’, multi-vesicular exocytosis and compound exocytosis (mass degranulation), depending on the amount of intracellular Ca^2+^ and type of activation (83, 84).

Combinations of IgE and substance P stimulation can result in either very localized (piecemeal) or systemic (compound) degranulation of MCs. Piecemeal exocytosis is related to complement factors C3a and C5a, endothelin and, most importantly, substance P (83). C3a and C5a are chemo-attractants for MCs in allergy and result in a rapid release of intracellular Ca^2+^ when activating MCs (85), which also has been observed in rejection (74).

After mast cell degranulation, proinflammatory cytokines as TNF-α, IL-6 and IL-8 are rapidly released. These cytokines also contribute to the inflammatory process as described in the following sections. In contrast, inhibition of IgE-dependent mast cell activation can be achieved by the cytokine TGF-β can inhibit mast cell degranulation and TNF-α production (86).

#### 2.2.2 Mast cells and pro-inflammatory pathways

MCs can produce and release pro-inflammatory cytokines upon various different stimuli. IgE stimulates MCs to release TNF-α, a pro-inflammatory cytokine, resulting in the recruitment of innate immune cells like neutrophils (25, 87), DCs and T cells. Mouse models have shown that after MC degranulation, histamine and serotonin increase vascular permeability (88, 89). In human models, MCs have been shown to also selectively release vascular endothelial growth factor (VEGF) together with IL-6 and IL-8 (90, 91). This combination can increase local vascular permeability and stimulate leukocyte and lymphocyte infiltration, which can result in a transplant can result in transplant dysfunction, due to rejection. Indirect communication with other innate cells occurs when MC granules are ingested by DCs and macrophages (25, 34).

#### 2.2.3 Crosstalk with T cells

Activated MCs primed with IgE can interact with various T cells, mainly through MHC-TCR interaction with co-stimulation of OX40L-OX40. TNF-α upregulates OX40L expression by MCs, and it is a potent factor in MC-T cell interaction (92). When linked with CD4+ Th cells, (co-)stimulation of TNF-α, IL-6 and MHC II antigen presentation will result in activation and proliferation of Th cells and release of pro-inflammatory cytokines (35, 93). In MC cross-talk with CD8+ T cells, (co-)stimulation with CCL5, 4-1BBL, TNF-α and MHC I antigen presentation will result in CD8+ recruitment, activation, proliferation and cytokine release (93, 94). While the OX40L-OX40 interaction activates T cells, it inhibits MC degranulation (94). Treg cells react differently to IgE activated MCs compared to CD4+ and CD8+ T cells; MCs suppress Treg activity through OX40L-OX40 cross-linking, in combination with histamine and IL-6 release (93). The crosstalk with T-cells, and in particular with CD8+ T cells can result in the development of an acute t cell-mediated rejection in the KTx as it is known that CD8+ T cells are a main player in transplant rejection (95).

#### 2.2.4 Crosstalk with B cells

MC interaction with B cells has been described in mice after migration of MCs from the skin to a draining lymph node (96). It is there where proliferation of B cells is achieved by OX40-OX40L as well as CD40-CD40L interaction in combination with MC derived IL-6 stimulation after IgE sensitization (97, 98). While most of these pathways lead to B cell activation and IgA, IgE or IgG producing plasma cells, pathways leading to IL-10 producing regulatory B cells have also been suggested (96, 98). It is in antibody mediated rejection that B-cells have a prominent function and the role of MC crosstalk with B-cells should also be further studied in this context (99).

### 2.3 Mast cells and allograft tolerance

MC tolerance to a specific antigen can be accomplished in several ways. Treg-MC interaction through OX40L-OX40 and IL-9 induces a tolerogenic state in MCs and perhaps the entire allograft (71, 94, 100, 101), provided the MC is not activated by IgE. OX40L activation in MCs will inhibit IgE-mediated degranulation. Tregs increase intracellular cAMP in MCs, resulting in lower levels of Ca^2+^, further inhibiting degranulation (102). IL-9, secreted by Tregs, Th9 and Th17 cells, regulates MCs, promoting their immune-suppressive functions and decreasing pro-inflammatory release (103). MCs produce IL-10 and TGF-β, which enhances Treg differentiation and recruitment, subsequently promoting Foxp3 expression (37, 94, 100). MCs secrete GM-SCF and TNF-α, resulting in a tolerogenic state of DCs (37, 104). In turn, tolerogenic DCs (tDCs) also increase tolerance through Treg proliferation, again through IL-10 and TGF-β (105). Although TNF-α is considered pro-inflammatory, it also enhances tolerance trough tDC stimulation (37). MC mediators that inhibit effector T cell proliferation and function include Mast Cell Protease 6 (MCP6), a tryptase inhibiting the pro-inflammatory IL-6 cytokine and Th7 cells (106), TGF-β, IL-10 (94, 107), and histamine (37). IL-10 and TGF-β induce anergy of naïve CD4+ and CD8+ T cells, or T cells cross-linked to APCs (108). Together with DCs, MCs can induce type 1 regulatory T cells (Tr1), which are immunosuppressive cells similar to Tregs (108, 109). Tr1s show suppressed alloreactivity to specific antigens and inhibit other naive alloreactive CD4+ T cells (110) by producing IL-10 and TGF-β themselves (108). TGF-β and IL-10 also inhibit FcϵRI function, implying MC self-regulation and DC inhibition of MC degranulation (86, 111). MC-derived IL-10, in co-stimulation with IL-4, results in suppression of progenitor MC recruitment and survival, thereby countering positive feedback loops of MC recruitment (112). IL-10 has anti-fibrotic capabilities (64), and together with tDCs, Tregs and Tr1s, MCs thus potentially modulate inflammation and fibrogenesis in KTx (113, 114).

IgE-mediated MC degranulation inhibits peripheral tolerance in multiple ways: the balance between effector T cells and Tregs is distorted, alloreactivity within T cells is restored and an efflux of Tregs out of the Tx is observed (115). Thus, MC degranulation in tolerant transplants can theoretically promote T cell-mediated rejection. It is important to note that even local degranulation can lead to systemic breakdown of peripheral tolerance.

### 2.3 Mast cells and fibrosis

Stressed or injured epithelial cells (e.g. due to hypoxia) can acquire a mesenchymal phenotype, a process known as epithelial-to-mesenchymal transition (EMT). In the kidney this process has been controversial, and most recently has been defined as partial EMT. The latter indicates mesenchymal transition of epithelial cells that do not become myofibroblasts but are important drivers of inflammation and fibrosis through cross-talk with immune cells and mesenchyme (116).

EMT is linked to myofibroblast activation and proliferation, Smad pathway activation and IF in the kidney, both dependently and independently of TGF-β (117, 118). Myofibroblasts originate from both fibroblasts and pericytes, and express α-SMA and high amounts of extracellular matrix upon activation of various pathways including but not limited to TGF-β, inflammatory, and extracellular matrix pathways (116).

An important factor in tissue TGF-β synthesis is the renin-angiotensin system (RAS) and its end-product angiotensin II (ANG II) (9). Angiotensin converting enzyme (ACE), mostly derived from lung capillaries, is required for conversion of ANG I to ANG II. MC-derived chymase is capable of cleaving ANG I, leading to an ACE-independent ANG II and subsequent TGF-β formation. Thus, kidney resident MC_TCs_ can contribute to intra-renal ANG II, TGF-β synthesis and fibrosis (119). MCs are also capable of releasing TGF-β as well as fibroblast growth factor-2 (FGF-2) (35, 120, 121). MMP-9, an ECM degrading enzyme secreted by MCs (also known as gelatinase B) (122), is another source of matrix-bound TGF-β activation and fibroblast contraction, further increasing MC potential to activate (myo)fibroblasts independently of RAS. Chymase can activate the plasmin system (123) and degrade fibrin/fibrinogen (124), thus countering the pro-fibrotic actions of the coagulation system. MCs have also been shown to crosstalk and form adhesion with tissue fibroblasts through c-KIT and CADM1 receptors. Crosstalk in combination with tryptase secretion leads to mostly pro-fibrotic activation and enhanced MC survival in co-culture studies, although select MC cultures exhibit anti-fibrotic activities (19).

Studies investigating MCs and fibrosis in human KTx patients are rare, but one study found a pro-fibrotic role of MCs, especially chymase positive MCs (44). Mouse models investigating MC influence on fibrosis shows the relationship to be more complex. Investigations using MC deficient mice show increased amount of fibrosis in aminonucleoside-nephrosis (125) and the unilateral ureteral obstruction model (126).

While MCs are regarded as inflictors of tissue fibrosis, MCs are also capable of modulating tissue remodeling. Local IL-10 release reduces collagen I deposition and decreased α-SMA and other fibroblast gene expression (127). Besides promoting fibroblasts, chymase also activates MMP-1 and MMP-3 function, both remodeling factors that degrade collagen fibers. Additionally, chymase cleaves and inactivates a latent factor called tissue inhibitor of metalloproteinase (TIMP-2), which inhibits MMP-2 within the ECM (128). MCs can express MMP-2 and MMP-9 themselves (122, 128, 129) after T cell mediated TNF-a stimulation (122). MCs are also capable of secreting, activating and removing inhibition of MMPs within the tubulo-interstitial compartment, a function unique to MCs. The contributory role of MMPs in fibrosis is complex, as e.g. TGF-ß increases both MMP-2 expression and release of its antagonist TIMP-2 (130).

## 3 Discussion

Mast cells are a pluripotent cell type that can either enhance or resolve injury, depending on their real-time environment. In this manuscript we propose a model depicting the multifaceted contribution of mast cells in the setting of kidney transplantation, namely in tolerance, rejection and chronic damage/fibrosis.

**Figure 1:**
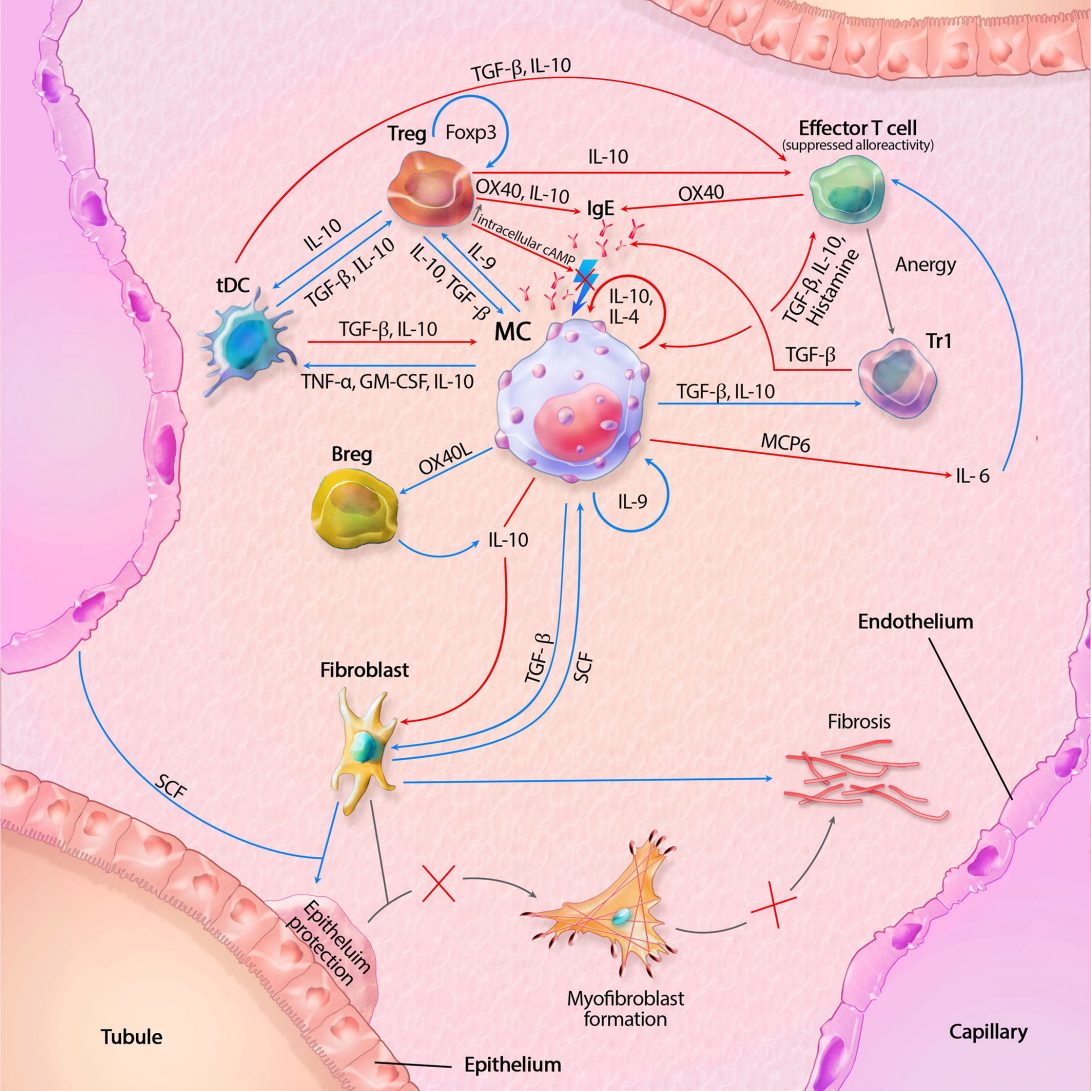
Mast cell (MC) interactions within the transplant during tolerance. FcϵRI activity is inhibited by TGF-β, IL-10 and OX40 ligation. Tregs also inhibit degranulation by lowering intracellular Ca^2+^ levels through increased cAMP. IL-10 suppresses alloreactivity within CD4+ and CD8+ T cells and promote anergy and regulatory functions of CD4+ T cells. IL-10 mediated inhibition of fibroblasts also inhibit subsequent formation of myofibroblasts. IL-10 with co-stimulation of IL-4 decrease MC proliferation, while IL-9 increases proliferation. GM-CSF, granulocyte-macrophage colony-stimulating factor; IL, interleukin; MCP6, mat cell protease 6; SCF, stem cell factor; tDC, tolerogenic dendritic cell; TGF-β, tissue growth factor beta; TNF-α, tissue necrotic factor alpha; Tr1, regulatory T cell type 1 (induced); Treg, regulatory T cell (natural); Blue lines symbolize activating pathways, red lines inhibitory pathways, gray lines symbolize subsequent events. Lighting icons are used in the most profound activation patterns, which are inhibited in tolerogenic environments.

**Figure 2:**
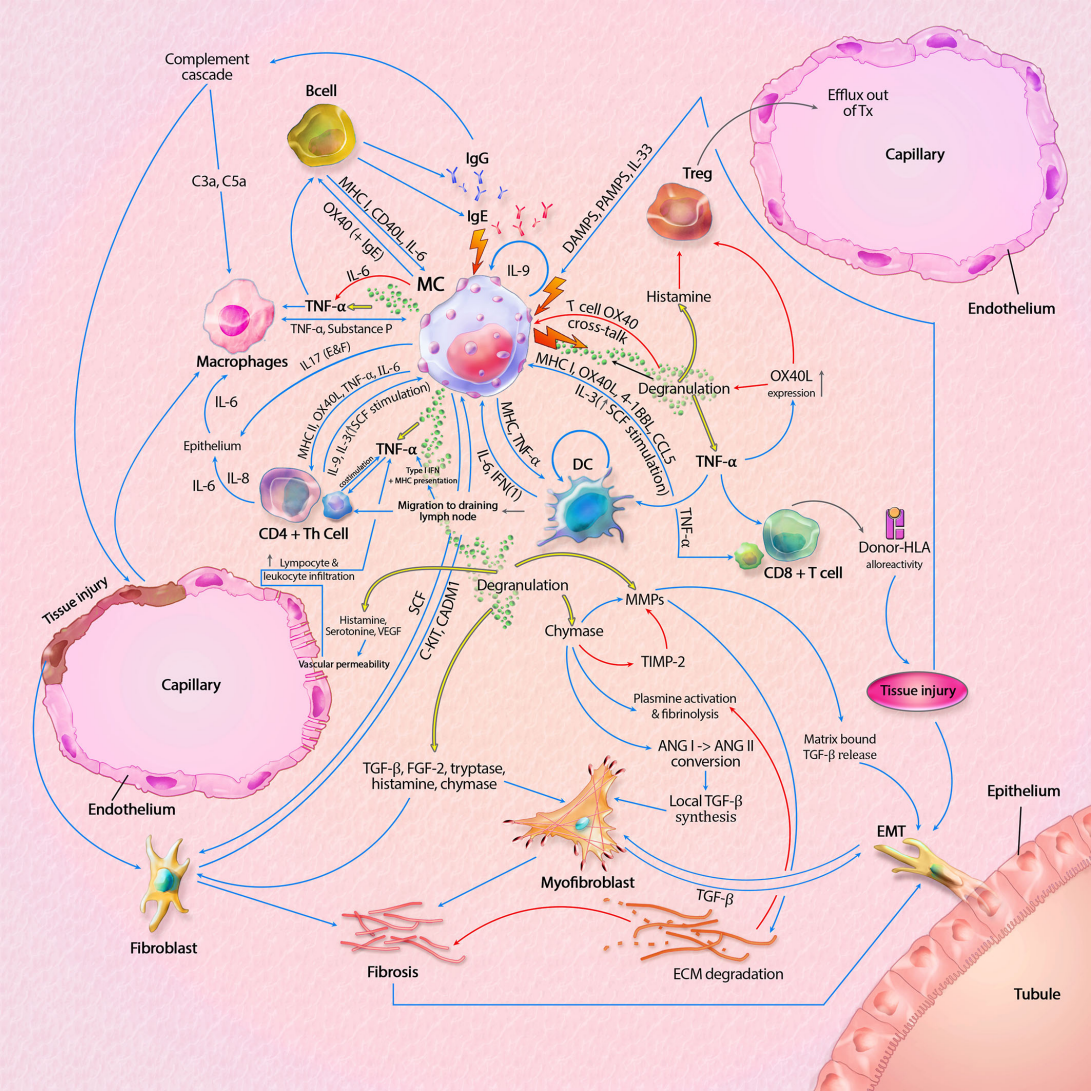
Mast cell (MC) interactions within the graft during rejection. Pathways can include both cytokines (like TNF-α) and membrane bound interaction (like MHC I-TLR interaction). MC-T cell interaction through OX40L-OX40 cross-linking inhibits MC degranulation, represented by the inhibitory pathway towards degranulation. Innate immune cells can also result in tissue injury, which is not shown in this model. Interaction between APCs, T cells and B cells, resulting in antigen production is also not shown in this model. The model shows almost no inhibitory pathways, explaining the progressive state of fibrosis within KTx even when immunosuppressive drugs are taken. Detailed description of the model can be found within the text. ANG, angiotensin; C3a/C5a, complement component; ECM, extracellular matrix; EMT, epithelial-mesenchymal transition; FGF-2; fibroblast growth factor-2; Ig, immunoglobulin; IL, interleukin; MHC, major histocompatibility complex; MMPs, matrix metalloproteinase; SCF, stem cell factor; tDC, tolerogenic dendritic cell; TGF-β, tissue growth factor beta; Th cell, T helper cell; TIMP-2, tissue inhibitor of metalloproteinase-2; TNF-α, tissue necrotic factor alpha; Treg, regulatory T cell (natural); VEGF, Vascular Endothelial Growth Factor. Blue lines symbolize activating pathways, red lines inhibitory pathways, yellow lines represent pre-formed mediators within MCs. Grey lines represent subsequent events. Lighting icons are used in the most profound activation patterns.

As discussed in this mini review, MCs contain a vast set of mediators and can act independently or in interaction with locoregional (immune) cells. There are distinct pro-inflammatory and tolerogenic patterns of interaction, with modulation of fibrogenesis. In kidney transplantation, IgE-mediated activation leads to the most profound degranulation, resulting in activation of pro-inflammatory and pro-fibrotic pathways, but IgE-independent activation also occurs. A hypothetical model, split between MC actions in rejection and transplant tolerance, is shown in **Figures 1**, **2**. This model portrays the most important pathways of all MC actions within the transplant.

Ultimately, the current model may constitute a paradigm shift: stimulating donor-tolerance should be considered, rather than focusing on immunosuppressive drugs, undermining the patient’s immune system (131). This would lessen the (therapeutic) burden of transplant recipients, could potentially prevent transplant rejection and would result in a more natural state of self-induced tolerance. Treg-based therapies are already being investigated, although long-term stability of said tolerance is unknown (3, 132). As our model shows, MCs could play an important role in inducing and upholding this state of tolerance towards the KTx. So, rather than eradicating or fully inhibiting MCs, MC modulation toward tolerogenic action should be investigated.

## Author contributions

MC-VG and TB conceptualized the review topic. GE gathered and summarized the relevant literature. GE and HV wrote the initial manuscript and TB, CB, JD-VH, MH, DH, RK, MR, MR, JT and MC-VG critically revised the manuscript. All authors contributed to the editing and finalization of the manuscript and approved the submitted version.
