# Corrigendum: The mast cell: A Janus in kidney transplants

**DOI:** 10.3389/fimmu.2023.1183969

**Published:** 2023-03-21

**Authors:** G. van der Elst, H. Varol, M. Hermans, C. C. Baan, J. P. Duong-van Huyen, D. A. Hesselink, R. Kramann, M. Rabant, M. E. J. Reinders, J. H. von der Thüsen, T. P. P. van den Bosch, M. C. Clahsen-van Groningen

**Affiliations:** ^1^ Department of Pathology and Clinical Bioinformatics, Erasmus University Center Rotterdam, Rotterdam, Netherlands; ^2^ Department of Internal Medicine, Division of Allergy and Clinical Immunology, Erasmus University Medical Center Rotterdam, Rotterdam, Netherlands; ^3^ Department of Internal Medicine, Division of Nephrology and Transplantation, Erasmus University Medical Center Rotterdam, Rotterdam, Netherlands; ^4^ Department of Pathology, Necker Hospital, APHP, Paris, France; ^5^ Institute of Experimental Medicine and Systems Biology, RWTH Aachen University, Aachen, Germany; ^6^ Division of Nephrology and Clinical Immunology, RWTH Aachen University Hospital, Aachen, Germany

**Keywords:** mast cell (MC), kidney transplant, rejection, fibrosis, tolerance

In the published article, there was an error in the legend for **Figures 1**, **2** as published. The legends of **Figures 1**, **2** were switched. The corrected legend appears below.

**Figure 1:**
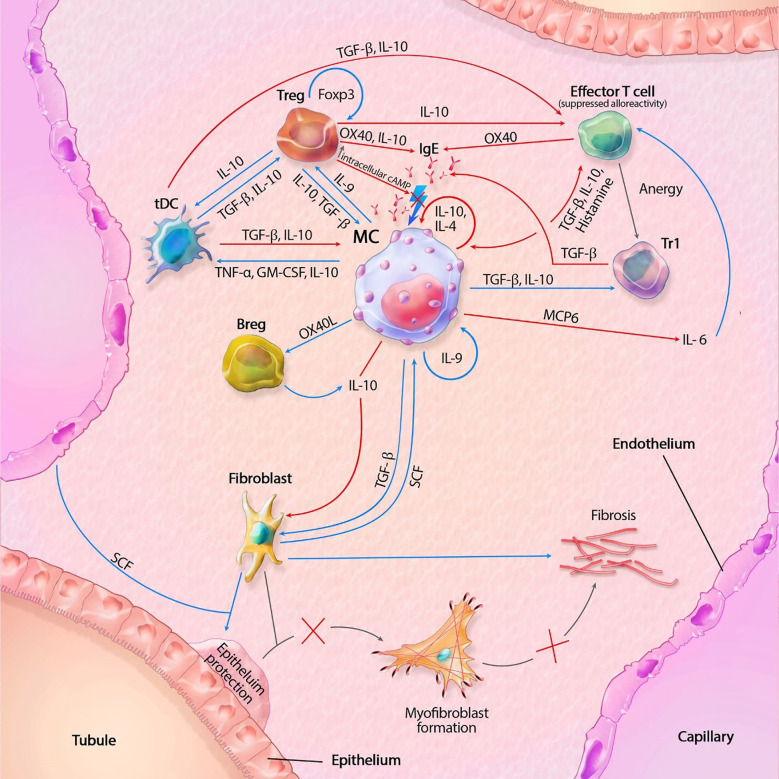
Mast cell (MC) interactions within the transplant during tolerance. FcϵRI activity is inhibited by TGF-β, IL-10 and OX40 ligation. Tregs also inhibit degranulation by lowering intracellular Ca^2+^ levels through increased cAMP. IL-10 suppresses alloreactivity within CD4+ and CD8+ T cells and promote anergy and regulatory functions of CD4+ T cells. IL-10 mediated inhibition of fibroblasts also inhibit subsequent formation of myofibroblasts. IL-10 with co-stimulation of IL-4 decrease MC proliferation, while IL-9 increases proliferation. GM-CSF, granulocyte-macrophage colony-stimulating factor; IL, interleukin; MCP6, mat cell protease 6; SCF, stem cell factor; tDC, tolerogenic dendritic cell; TGF-β, tissue growth factor beta; TNF-α, tissue necrotic factor alpha; Tr1, regulatory T cell type 1 (induced); Treg, regulatory T cell (natural); Blue lines symbolize activating pathways, red lines inhibitory pathways, gray lines symbolize subsequent events. Lighting icons are used in the most profound activation patterns, which are inhibited in tolerogenic environments.

**Figure 2:**
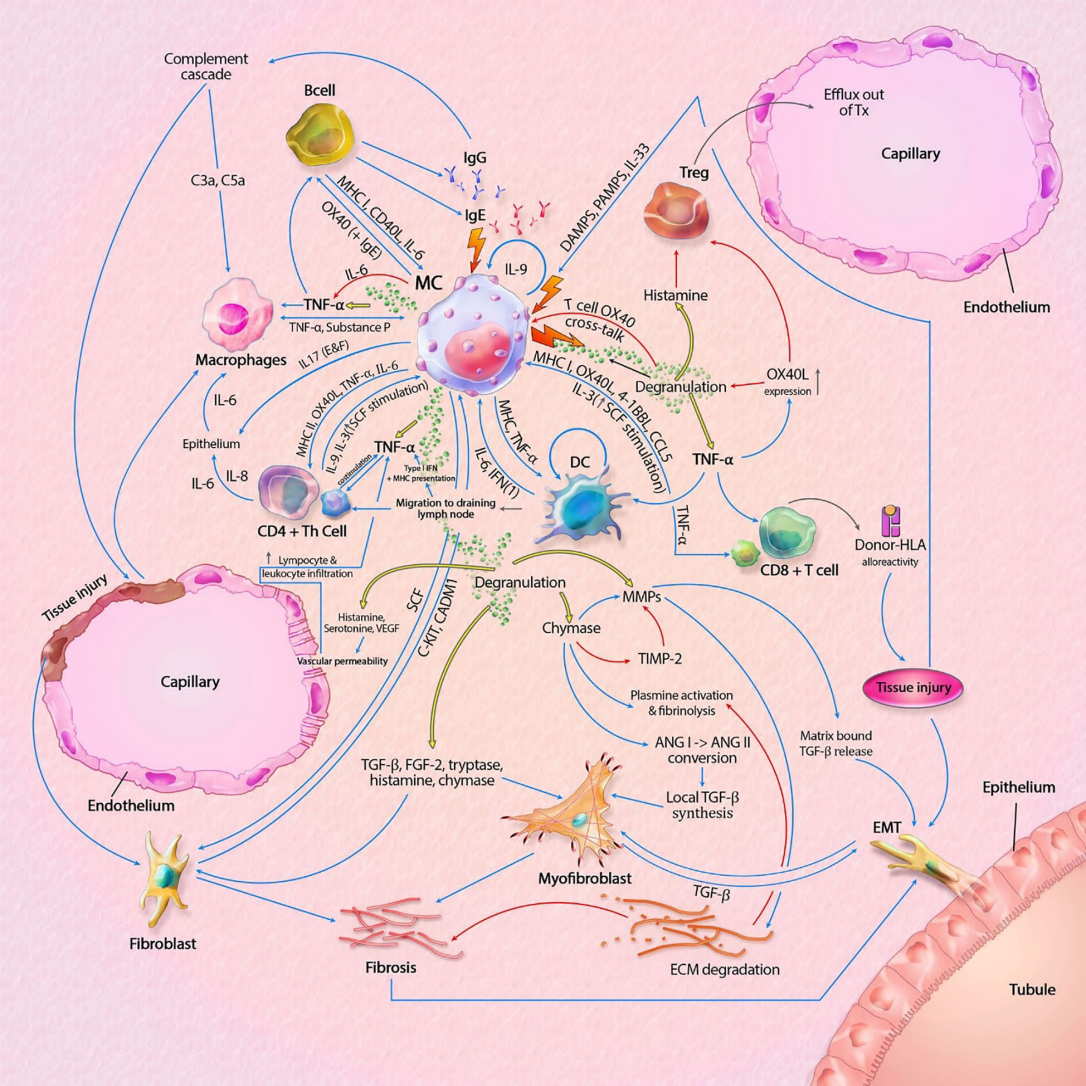
Mast cell (MC) interactions within the graft during rejection. Pathways can include both cytokines (like TNF-α) and membrane bound interaction (like MHC I-TLR interaction). MC-T cell interaction through OX40L-OX40 cross-linking inhibits MC degranulation, represented by the inhibitory pathway towards degranulation. Innate immune cells can also result in tissue injury, which is not shown in this model. Interaction between APCs, T cells and B cells, resulting in antigen production is also not shown in this model. The model shows almost no inhibitory pathways, explaining the progressive state of fibrosis within KTx even when immunosuppressive drugs are taken. Detailed description of the model can be found within the text. ANG, angiotensin; C3a/C5a, complement component; ECM, extracellular matrix; EMT, epithelial-mesenchymal transition; FGF-2; fibroblast growth factor-2; Ig, immunoglobulin; IL, interleukin; MHC, major histocompatibility complex; MMPs, matrix metalloproteinase; SCF, stem cell factor; tDC, tolerogenic dendritic cell; TGF-β, tissue growth factor beta; Th cell, T helper cell; TIMP-2, tissue inhibitor of metalloproteinase-2; TNF-α, tissue necrotic factor alpha; Treg, regulatory T cell (natural); VEGF, Vascular Endothelial Growth Factor. Blue lines symbolize activating pathways, red lines inhibitory pathways, yellow lines represent pre-formed mediators within MCs. Grey lines represent subsequent events. Lighting icons are used in the most profound activation patterns.

The authors apologize for this error and state that this does not change the scientific conclusions of the article in any way. The original article has been updated.

